# Comparison of the effects of *Aloe vera* gel and coconut oil on the healing of open wounds in rats

**DOI:** 10.17221/101/2021-VETMED

**Published:** 2023-01-13

**Authors:** Ozmen Istek, Murat Tanrisever, Sefa Kucukler, Burak Karabulut, Aydin Cevik

**Affiliations:** ^1^Department of Nursing, Faculty of Health Sciences, Mus Alparslan University, Muş, Türkiye; ^2^Department of Surgery, Veterinary Faculty, Firat University, Elazığ, Türkiye; ^3^Department of Biochemistry, Veterinary Faculty, Atatürk University, Erzurum, Türkiye; ^4^Department of Pathology, Veterinary Faculty, Firat University, Elazığ, Türkiye

**Keywords:** *Aloe vera* gel, coconut oil, rat, wound healing of animals

## Abstract

In this study, the effects of *Aloe vera* gel and coconut oil on wound healing were investigated and compared in rats. Forty-two Wistar albino rats were used during the experiment, in which they were operated on under general anaesthesia to create two full-thickness open skin wounds (created with a 0.5 cm diameter punch biopsy apparatus) on both back sides of the median line. A total of 42 rats were divided into three groups of 14 animals each to receive the topical application of *Aloe vera* gel (AV group – *n* = 14), coconut oil (CO group – *n* = 14) and cold cream (CONT group – *n* = 14). The medical applications were performed twice a day in all the groups. The wound borders were marked on a transparent sheet every day. Afterwards, this sheet was transferred to the millimetre graph paper. On days 0, 7, and 14, the unhealed wound area was measured in all the groups. On days 7 and 14, seven rats in each group were euthanised. Then, skin samples including the intact skin were taken from the wound sites for histopathological and biochemical evaluations. The topical application of *Aloe vera* gel showed a significant increase in the healing process of the open wounds in terms of the clinical evaluation, histopathological and biochemical data averages when compared with the coconut oil and cold cream groups of rats (*P* < 0.05). The results obtained in the present study demonstrate that *Aloe vera* gel may provide a good alternative for the treatment of open wounds.

A wound is defined as the disruption of the anatomical and functional continuity of living tissue (Nguyen et al. 2009). Wounds are classified as acute and chronic wounds. Acute wounds are those in which the healing is mostly normal, adequate anatomical and functional integrity is provided and the repair process takes place at the prescribed time (Lazarus et al. 1994). This sequence of events is the process from inflammation, collagen and fibroblast deposition, angiogenesis to wound contraction and scar remodelling. On the other hand, chronic wounds are those in which the repair is impaired (infection, immunosuppression, etc.), which delays the healing process (Evans 1980; Li et al. 2007; Kumar et al. 2014). The post-injury healing phenomenon occurs in five stages; inflammation, neovascularisation, granulation tissue formation, reepithelisation of the new extracellular matrix and tissue remodelling (Evans 1980; Nguyen et al. 2009; Rieger et al. 2015). Since ancient times, humans have used various parts of plants for prophylactic and therapeutic purposes against diseases including wounds and burns (Principe 2005; Chah et al. 2006; Alam et al. 2011). It has been reported that grape seed oil, which is rich in phenolic compounds, free fatty acids and vitamins, improves the rate of wound closure and shortens the healing process (Nayak et al. 2011). It has been emphasised that compounds including polyphenols, tocopherols, sterols, squalene, and triterpene alcohols (Boucetta et al. 2015), along with argan oil are effective in the treatment of burn wounds (Avsar et al. 2016). It has been reported that turmeric, whose main component is curcumin, increases granulation tissue formation, collagen deposition, tissue remodelling, and wound contraction, thereby shortening the healing process of the wound (Panchatcharam et al. 2006; Madhyastha et al. 2010). It has been reported that after being topically applied to the surgical wounds of experimental animals, calendula, containing triterpendiol esters, saponins, and flavonoids, leads to faster epithelialisation and increases fibroblast proliferation (Fronza et al. 2009; Preethi and Kuttan 2009). Avocado oil is rich in linoleic acid, linolenic acid, oleic acid, β-sitosterol, β-carotene, lecithin, minerals, and vitamins such as A, C, D and E (de Oliveira et al. 2013). It has been shown in previous studies that when applied topically, avocado oil improves the collagen synthesis, increases the epithelialisation, and reduces the number of inflammatory cells during the wound healing process in rats (Nayak et al. 2008; de Oliveira et al. 2013). Today, synthetic drugs used in the treatment of wounds are expensive and seem to cause allergic problems, drug resistance, etc. Thus, scientists are forced to look for alternative drugs (Chithra et al. 1998; Cohen et al. 2000; Fabricant and Farnsworth 2001). Research studies on wound healing have revealed that herbal compounds should be used rather than chemical substances (Somboonwong et al. 2000).

*Aloe vera* is a tropical succulent plant that is a member of *Liliaceae-Lily* family. The gel obtained from the plump leaves of the plant has been used for medical purposes since ancient times (Cohen et al. 2000). The effect of *Aloe vera* on wound healing is due to glucomannan and mannose which are rich in polysaccharides. In addition, growth hormones, such as gibberellin, interact with growth factor receptors on the fibroblast, thereby stimulating its activity and proliferation, which in turn significantly increases the collagen synthesis of *Aloe vera* (Mantle et al. 2001).

Coconut (*Cocos nucifera* L.) belongs to the family of *Arecaceae* (*palmae*), a sub-family named *Cocoaeae*. Young coconut oil is widely used in food and medicinal industry (Satheesh and Prasad 2012). Chemically, coconut oil contains polyphenols, medium-chain unsaturated fatty acids, biologically active ingredients and sterols (Fife 2003; DebMandal and Mandal 2011). Topical applications of coconut oil on wounds have been reported to stimulate collagen production, thereby accelerating wound healing. A study of Nevin and Rajamohan’s (2010) showed that coconut oil has a significant positive effect on wound repair by accelerating the epithelialisation process in wound healing. In this study, we aimed to investigate and compare the effects of *Aloe vera* gel and coconut oil on wound healing in rats.

## MATERIAL AND METHODS

### Chemicals

The *Aloe vera* plant gel was extracted from *Aloe vera* leaves obtained from a flower shop, the coconut oil was obtained from Talya Herbal LLC (Antalya, Türkiye) and the cold cream which, is an emulsion of water and certain fats (Botafarma, 12.5% spermaceti + 12% white wax + 56% liquid paraffin + 0.5% borate of soda + 19% distilled water), was obtained from Galenik Pharmacy (İzmir, Türkiye). Xylazine hydrochloride (Rompun, Bayer, Türkiye) and ketamine hydrochloride (Ketalar, Parke-Davis, Türkiye) were used for anaesthesia in rats. In addition, acetaminophen (Calpol, GlaxoSmithKline, Türkiye) was used as a post-surgical analgesic (2 mg/ml) in the rats’ daily drinking water.

### Animals

In this research, forty-two female Wistar albino rats (two months old; 250–260 g) were used. They were kept in a 12 h light/dark cycle, at a temperature of (24 ± 1 °C) with constant humidity (45 ± 5%). The animals were kept in separate cages until euthanasia.

### Experimental protocol

This study was conducted with the approval of the Animal Experiments Ethics Committee of Firat University (Law No. 147, dated 2018, September 5).

### Wound formation

The rats were anaesthetised intramuscularly with 6 mg/kg xylazine hydrochloride (Rompun^®^; Bayer, Bayer, Türkiye) and 85 mg/kg ketamine hydrochloride (Ketalar^®^; Pfizer, Parke-Davis, Türkiye).

Two full-thickness open skin wounds were created with a 0.5 cm diameter punch biopsy apparatus on both back sides of the median lines after the hair on the dorsum was clipped widely from the scapula to the ilium region and the clipped area was surgically prepared with polyvidone-iodine (Betadine^®^; Kansuk, Istanbul, Türkiye) and each rat was positioned in sternal recumbency and surgically draped.

### Treatment protocol

The *Aloe vera* gel was applied to the AV group rats, the coconut oil was applied to the CO group rats and the cold cream was applied to the CONT group rats, topically twice a day. The wound borders were marked on a transparent sheet every day.

Observations during the daily wound care: Each wound was evaluated daily for wound healing and the presence of any exudate until day 14. The day that the first granulation tissue was observed and the days that the wound was filled with granulation tissue and covered with epithelialised cells were recorded.

### Evaluation of wound healing

In terms of the physical features of the wounds, the wound areas marked on the transparent paper on days 0, 7, and 14 were calculated by the number of squares covered by the wound area transferred from transparent paper to millimetre graph paper. The degree of the wound closure to the original wound site was calculated as a percentage using the Walker and Mason (1968) formula (Rieger et al. 2015).

### Histopathological examination

The skin samples containing the wound site were fixed in a 10% neutral formalin solution for 48 hours. The prepared sections were stained with haematoxylin and eosin and Masson’s trichrome staining in an automatic tissue dyeing machine (Leica Autostainer XL; Leica, Wetzlar, Germany). The examined wound sites were scored: semiquantitatively in terms of inflammation, ulceration, vascularisation, surface closure, fibrosis, and regeneration of the dermis components, such as hair follicles and sweat glands (0 – none, 1 – mild, 2 – moderate, 3 – severe).

### Biochemical examination

The malondialdehyde (MDA) levels in the skin homogenate were measured using the thiobarbituric acid reaction according to the method performed by Placer et al. (1966) and expressed as nmol/g tissue. The skin catalase (CAT) activity was measured according to the method made by Aebi (1984) and was determined as kat/g protein. The protein concentration of the supernatant was measured according to the method of Lowry et al. (1951). The glutathione (GSH) level was measured according to the Sedlak and Lindsay (1968) method and expressed as nmol/g skin tissue. The glutathione peroxidase (GPx) activity was defined according to the method performed by Matkovics et al. (1988) and expressed as IU/g protein. The superoxide dismutase (SOD) activity was measured according to the method of Sun et al. (1988). This activity measurement is based on nitroblue tetrazolium (NBT) degradation of the superoxide radical produced by the xanthino-xanthine oxidase system and formazan at 560 nm.

### Statistical analysis

The sample size of the study was determined according to the GPower v3.1 (Heinrich Heine University, Germany) power analysis software. The power of the *F* test was chosen as 0.80 by using the software. Due to considering the ethical restriction in the rats, the “effect size *f*” value was adjusted as “small” (Faul et al. 2007). The biochemical data were analysed by a one-way analysis of variance (ANOVA) and the wound healing parameters were analysed with the Kruskal-Wallis *H* test using the SPSS (v25.0; IBM, USA) statistical program. All the values were expressed as mean ± standard error (SEM), and the results were considered significant at *P* < 0.05.

## RESULTS

### Findings of wound healing

**Figure 1:**
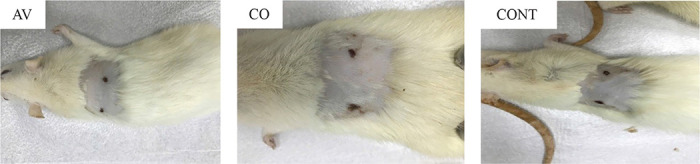
Progression of the wound healing in the AV (*Aloe vera*), CO (coconut oil) and CONT (control) groups on day 7

**Figure 2:**
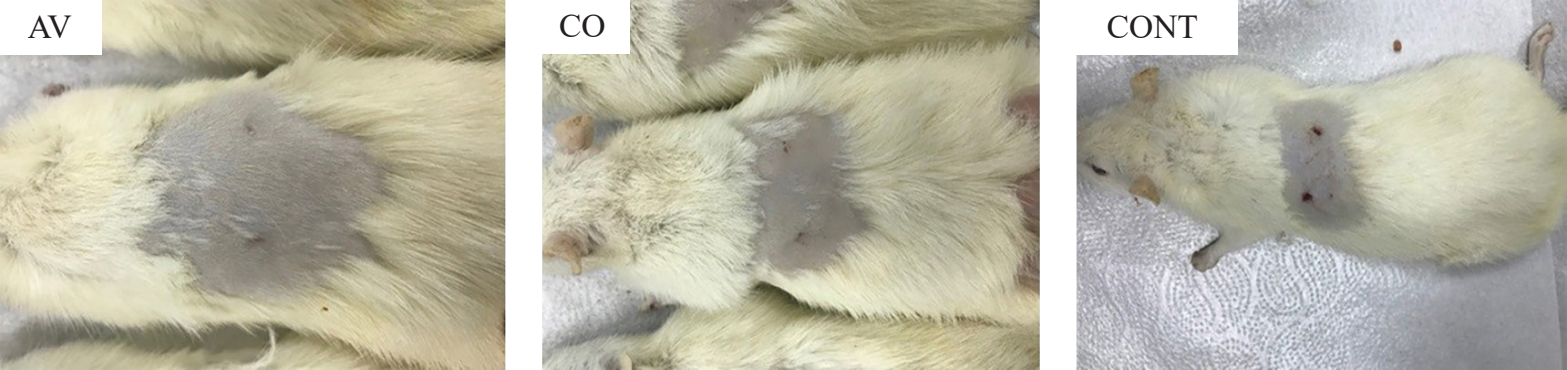
Progression of the wound healing in the AV (*Aloe vera*), CO (coconut oil) and CONT (control) groups on day 14

In this study, the macroscopically determined closure levels of the wounds on days 7 and 14 in the *Aloe vera* gel, coconut oil and cold cream groups are shown in Figures 1 and 2.

All the groups were evaluated for the mean levels of the unhealed wound areas and the parameters on days 7 and 14 according to the healing process as shown in Table 1.

**Table 1:** The calculation of the wound non-healed area in all the groups on days 0, 7 and 14

Days	*Aloe vera*	Coconut oil	Control
1^st^ day right	35.00 ± 0.00*	35.00 ± 0.00*	35.00 ± 0.00*
1^st^ day left	35.00 ± 0.00*	35.00 ± 0.00*	35.00 ± 0.00*
			
7^th^ day right	3.14 ± 0.89*	4.14 ± 1.06^♦^	4.14 ± 0.69^♦^
7^th^ day left	3.14 ± 0.89*	3.85 ± 1.34^♦^	4.71 ± 0.75^♦^
			
14^th^ day right	1.00 ± 0.00*	1.57 ± 0.53^♦^	2.14 ± 0.69^●^
14^th^ day left	0.85 ± 0.37*	1.42 ± 0.53^♦^	2.42 ± 0.78^●^

Values are expressed as the mean ± standard error mean; *P* < 0.05

*^,♦,●^Values pointed with a same symbol do no show difference of non-healed area

According to the daily wound observation of the rats: on the seventh day of the healing process, the wound healing in the AV group was better than the CO and the CONT groups. Considering the data obtained on day 14, it was determined that there was a more significant improvement (*P* < 0.05) in both wound areas of the AV group compared to the CO and CONT groups. Moreover, the wound healing in the wounds treated with coconut oil was observed to be better than the CONT group on the fourteenth day compared to the seventh day.

### Histopathological findings

**Figure 3:**
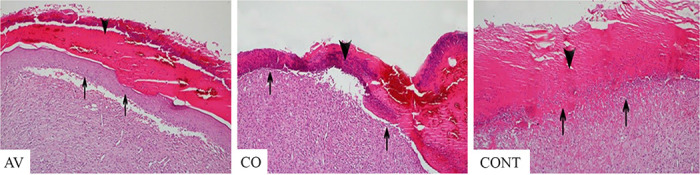
The sequestrum and ulceration (arrow heads) in the wound side in all the groups, can be seen in the AV group and cannot be seen in the CO and CONT groups, epithelisation (arrows) under the scar, on day 7 H&E staining; × 100 magnification AV = *Aloe vera*; CO** =** coconut oil; CONT** =** control

**Figure 4:**
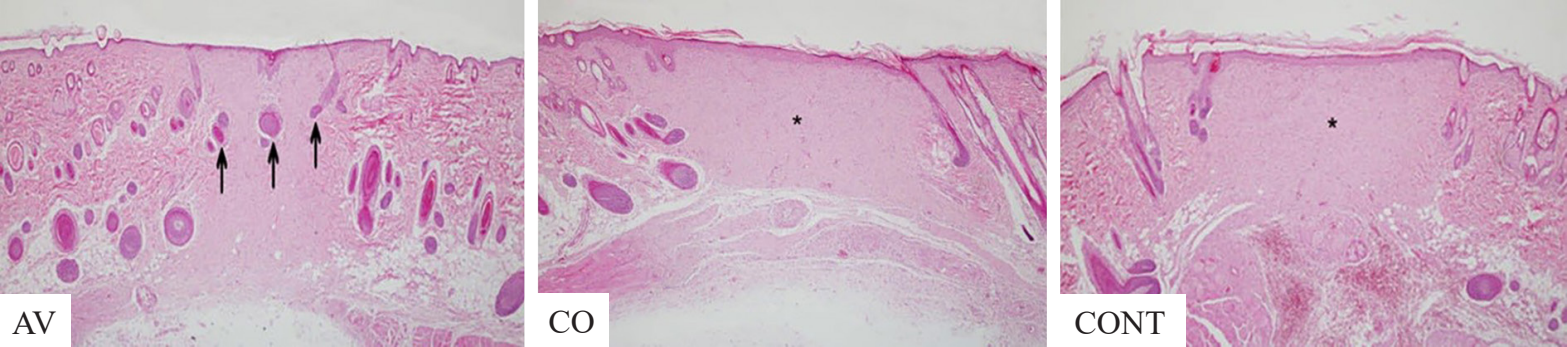
The hair follicle regeneration (arrows) started in the dermis (*) in the AV group only, on day 14 H&E staining; × 40 magnification AV = *Aloe vera*; CO** =** coconut oil; CONT** =** control

**Figure 5:**
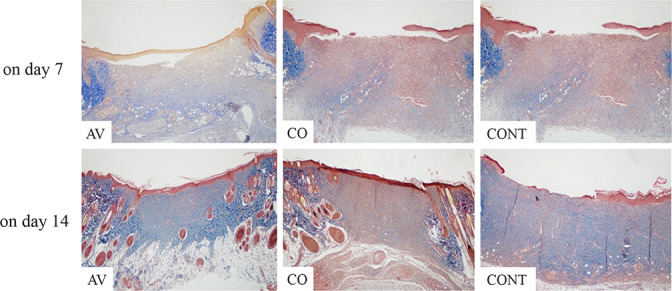
Fibrosis that develops in the wound healing process Masson’s trichrome staining; × 40 magnification AV = *Aloe vera*; CO** =** coconut oil; CONT** =** control

**Figure 6:**
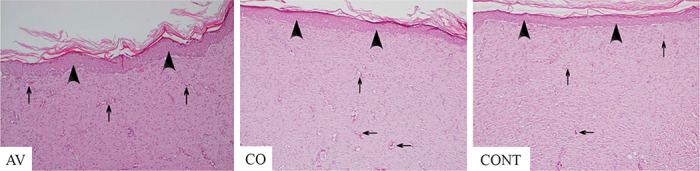
The epithelisation (arrow heads) and vascularisation (arrows) in the dermis in all the groups on day 14 H&E staining; × 100 magnification AV = *Aloe vera*; CO** =** coconut oil; CONT** =** control

On day 7, ulceration and scar formation were observed on the wound areas in all the groups (Table 2 and Figure 3). There was mild inflammatory infiltration and fibrosis in all the groups’ wound areas. Only the wound areas of the AV group began to close by epithelisation. Also, the hair follicles and sweat glands were seen only in the dermis tissues of the wound areas of AV group (Figure 4). Similarly, the vascularisation increased slightly in all the groups. On day 14, although all the group wounds were covered fully with granulation tissue and epithelialisation, the best epithelisation occurred in the AV group. The inflammation and fibrosis were similar in all the groups (Table 2 and Figure 5). The new hair follicle and sweat gland developments were seen only in the AV group. Giant cells were found in all the groups in the dermis tissues of the wound areas. It was observed that vascularisation on day 14 increased in all the groups compared to that on day 7. According to the data (Figure 6), there was an important difference among the groups with regard to the epithelisation process and surface closure, which were determined to be more rapid in the AV group compared to the two other groups.

**Table 2:** Histopathological scores on days 7 and 14

Parameters	On day 7^a^				On day 14^b^			*P*- value
	AV	CO	CONT		AV	CO	CONT	
Inflammation	+	+	+		+	+	+	0.027
Ulceration	+++	+++	+++		–	–	–	
Vascularisation	+	+	+		++	++	++	
Surface closure	+	–	–		+++	++	++	
Fibrosis	++	++	++		++	++	++	

^a,b^Within a row values with a different superscript show differences (*P* < 0.05)

AV = *Aloe vera*; CO** =** coconut oil; CONT** =** control

### Biochemical findings

When the biochemical data were analysed overall, a significant increase was observed in the AV group compared to the CONT group whereas the increase between the CO and AV groups was not significant (*P* < 0.05) (Table 3).

**Table 3:** GSH and MDA levels with the antioxidant enzyme activities in the healing processes of the wounds in all the groups

Groups	MDA	GSH	SOD	CAT	GPx
Day 7					
AV	12.15 ± 0.49^a^	3.18 ± 0.05^b^	9.04 ± 0.07^a^	2.84 ± 0.02^b^	7.38 ± 0.06^b^
CO	15.41 ± 0.35^b^	2.97 ± 0.03^b^	8.61 ± 0.10^b^	2.69 ± 0.02^b^	7.11 ± 0.05^b^
CONT	16.82 ± 0.4^b^	2.61 ± 0.03^a^	8.20 ± 0.06^b^	2.30 ± 0.03^a^	6.83 ± 0.04^a^
					
Day 14					
AV	10.03 ± 0.17^a^	3.67 ± 0.06^b^	9.71 ± 0.19^c^	3.34 ± 0.09^b^	9.17 ± 0.16^b^
CO	11.78 ± 0.41^a^	3.19 ± 0.04^a^	9.24 ± 0.16^b^	3.00 ± 0.04^b^	7.87 ± 0.09^a^
CONT	13.73 ± 0.48^b^	3.02 ± 0.04^a^	8.66 ± 0.05^a^	2.76 ± 0.04^a^	7.28 ± 0.07^a^

^a–c^Within a column values with a different superscript show differences (*P* < 0.05)

The above results are shown as the mean ± standard error of the mean (*n* = 7)

AV = *Aloe vera*; CAT = catalase; CO = coconut oil; CONT = control; GSH = glutathione; GPx = glutathione peroxidase; MDA = malondialdehyde; SOD = superoxide dismutase

Statistically, when the malondialdehyde (MDA) data of days 7 and 14 were compared, it was observed that it decreased significantly in the AV group but there was not any difference in the CO group. This decrease was significant in the AV and CO groups in comparison with the CONT group. In terms of the AV group, the reduction on day 14 was much more significant.

When the glutathione (GSH) levels were investigated, a significant increase was detected in the AV group, but a less effective increase was observed in the CO group (*P* < 0.05) as a positive effect. When the results of day 14 were examined, this effect was observed just in the AV group.

According to the findings related to superoxide dismutase (SOD); on the day 7, only the AV group score was significantly changed (*P* < 0.05). However, although the findings regarding the day 14 show that there was an increase in scores of both CO and AV groups, only the increases of AV group scores was statistically significant.

The average values of the catalase (CAT) functional protein increased in both the AV and CO groups, but it was more prominent in the AV group (*P* < 0.05). In addition, a significant increase in the CAT value continued on day 14 in the AV group; but it decreased in the CO group.

When the glutathione peroxidase (GPx) data were evaluated, there was a significant increase in the results of the AV group compared to those of the CONT group on day 7. However, there was a significant fluctuation in the CO group results. On day 14, it was observed that the effect in the AV group was more significant, but it was less in the CO group in terms of the GPx results (*P* < 0.05).

## DISCUSSION AND CONCLUSION

There are numerous reports on the effects of wound healing. When *Aloe vera* gel is used pure or as a formulated product, it elicits relatively less inflammation, more collagen, and angiogenesis in restoring cellular structures and tissue layers in damaged tissue (Davis et al. 1987; Miller and Koltai 1995; Visuthikosol et al. 1995). It has been stated that *Aloe vera* has healing effects, accelerates the epithelialisation along with neovascularisation, increases the wound contraction rate, and provides faster healing (Fulton 1990; Heggers et al. 1993). *Aloe vera* can also be considered as a highly economical therapeutic agent, as it can be used as an inexpensive and effective topical gel. The positive effect of the AV gel on wound healing in our study is consistent with these studies. Chithra et al. (1998) suggested that in wounds treated with *Aloe vera*, it had a positive effect on the proliferation phase, which affected the fibroplasia and collagen synthesis, thereby increasing the healing area.

Haritha Yadav et al. (2012) believed that *Aloe vera* gel may induce wound contraction and the healing process via promoting epithelialisation, and neovascularisation processes. Moreover, they observed that *Aloe vera* has a significant influence on the level of collagen which is the precursor protein for the wound healing mechanism. The data from our study with the AV gel (Tables 2 and 3) on the surface closure, vascularisation of the wounds, formation of dermal regeneration and shaping of the complete closure of wounds, present better results when compared with the other groups.

Batool (2012), in his study, reported a faster recovery rate in animals treated with coconut oil compared to the control group especially on day 14 of the treatment due to more collagen bundle formation and complete epithelialisation formation, resulting in a reduced healing time and faster healing. Referring to the data in Figures 5 and 6, it can be seen that on day 14, the rats’ wound healing findings were parallel with this study.

In a study investigating the wound healing efficacy of *Aloe vera* gel in an excision wound model, it was reported that the healing began to occur after the 4^th^ day as a result of contractions in the area where the excision was performed. There was a significant difference on the 14^th^ day of scar formation between the treated groups and, thus, *Aloe vera* gel was recommended for the treatment of traumatic or cut wounds (*P* < 0.01) (Takzare et al. 2009).

In our study, it was observed that on days 7 and 14 of the AV gel applications onto the wounds, there were positive changes in the wound areas as shown in Figures 1 and 2. Moreover, the statistical values in Table 1 show that our study exhibits similarities with this study.

In the coconut oil-treated wounds, with the shortening of epithelization time and high levels of various skin components, a much faster recovery was observed as well as a significant increase in the amount of collagen (Amro et al. 2018). In another study, the complete wound healing was achieved in rats’ wounds treated with coconut oil at the end of the 18^th^ day due to contractions on the wound area and it was also stated that coconut oil also supported this healing effect by preventing infection and prolonged inflammation (Wilson et al. 2015). In our study, the data obtained from the CO group supported the regeneration and epithelisation of the dermis components.

Subramanian et al. (2006) showed that subjects treated with *Aloe vera* made a good recovery on day 14 compared to the baseline. Additionally, there was a significant reduction in the wound size from day 4 till day 14 and the rate of wound closure was faster in this group compared to that in the CONT group. Also, well-developed granulation tissue organisation was noticed on day 7. Furthermore, after 14 days of the AV gel treatment, complete recovery and scar formation were observed in the animals in their study. The results obtained in Figures 1 and 2 regarding the wound healing processes on day 7 and 14 in our study are seen to be similar to these cases.

Nevin and Rajamohan (2010), in their study, reported an increase in the SOD, GPx and glutathione reductase (GR) activity in the wounds treated with coconut oil through topical applications. They also determined that the increase in the activity of the antioxidant enzymes did not lead to a decrease in the lipid peroxides (for example MDA) in the treated rats. On day 14, however, while the antioxidant enzymes returned to the basal levels, the SOD and GPx activities (Table 3) increased similarly to those of the above study.

According to another study, it was made verious sizes of full-thicknes wounds (1–2.25 cm^2^) on the animals’ backs. They found that the healed wound larger than 0.5 cm in diameter had new hairs formation at the centre of the wound compared to the smaller healed wounds (Chuong 2007).

Ito et al. (2007) fully described and characterised *de novo* hair follicle neogenesis that was dependent on the wound-induced hair follicle neogenesis (WIHN) signalling after a full-thickness wound in mice. The fact that hair follicles were only seen on the rats treated with *Aloe vera* gel in our study suggests that *Aloe vera* gel may induce the formation of hair follicles in the full-thickness wound healing of rats via a mechanism described by the former authors.

In conclusion, it is thought that the use of *Aloe vera* gel may be beneficial for the treatment of traumatic wounds on the skin and may induce the formation of hair follicles in full-thickness wound healing in rats, especially considering the physical, histopathological and biochemical findings of the present study. Moreover, it was concluded that further studies can be planned to show the effects of *Aloe vera* on the wound healing and hair regeneration.
